# Management of Mechanical Strabismus After Complicated Functional Endoscopic Sinus Surgery (FESS)

**DOI:** 10.3390/jcm14103360

**Published:** 2025-05-12

**Authors:** Katarzyna Pelińska, Justyna Simiera, Piotr Loba

**Affiliations:** Department of Binocular Vision Pathophysiology and Strabismus, Medical University of Lodz, Narutowicza Street 96, 90-141 Lodz, Poland; justynaolcz@gmail.com (J.S.);

**Keywords:** diplopia, endoscopic sinus surgery, restrictive strabismus, strabismus surgery

## Abstract

**Objectives:** Although considered a safe procedure, functional endoscopic sinus surgery (FESS) can cause various significant ophthalmic complications, i.e., serious extraocular muscle (EOM) damage. The aim of this study is to review the surgical management outcomes of patients with mechanical strabismus and diplopia as a complication of FESS, who referred to ophthalmological department in Norbert Barlicki University Teaching Hospital No. 1 over the 5-year period from 2018 to 2023. **Methods:** The records of seven consecutive patients with diplopia following endoscopic sinus surgery were retrospectively reviewed. Demographics, ophthalmological and orthoptic examination, the results of orbital imaging, type of FESS, type of strabismic surgery, and the timing of the first intervention were analysed. **Results:** The time from FESS to referral for strabismic intervention varied from one day to two months. Two patients, who were operated upon immediately after the FESS procedure, underwent direct reunion of the proximal and distal parts of the ruptured medial rectus muscle. One patient required maxillofacial intervention in order to improve prominent enophthalmos. The remaining five demonstrated severe adhesion formation around at least one of the EOMs and orbital walls. Only patients who were operated upon within a short period after complicated FESS achieved orthotropia and lack of diplopia in the primary position with a single surgery. **Conclusions:** Early recognition of the orbital complications subsequent to FESS and prompt referral are essential for achieving a satisfactory surgical result. Appropriate treatment should be based on the mechanism, location, type, and severity of muscle damage.

## 1. Introduction

Functional endoscopic sinus surgery (FESS) is one of the most frequently performed surgical procedures to restore physiological airflow and drainage through the paranasal sinuses in patients with chronic or recurrent rhinosinusitis [1,2]. This minimally invasive technique is also used to remove foreign bodies, polyps, and small cancerous tumors of the nose or sinuses, to decompress the orbit, resect pituitary tumors, and to treat obstruction or malformations of the lacrimal duct [3,4]. The growing number of indications and performed procedures entails an increased incidence of complications of FESS.

However, due to the proximity of the orbital walls and the paranasal sinuses, the orbital contents are at risk of direct injury if the orbit is inadvertently entered during the FESS procedure. The overall incidence of complications ranges from 0.031 to 3.1% [5]. Major complications are less common for primary FESS (0.36%) than for revision surgery (0.46%) [2]. Krings et al. reported major orbital complications occurring in 0.23% of cases in a cohort of nearly 80,000 patients [2]. They included retrobulbar hemorrhages, and injury to the optic nerve, the nasolacrimal duct, or the extraocular muscles [1,6]. Direct damage to one or several extraocular muscles and resultant restrictive strabismus leads to troublesome diplopia, abnormal head posture, and significant limitation of ocular motility. The management of these complications is challenging, usually requiring multiple surgeries, and desirable postoperative results are difficult to achieve.

The aim of this study is to review the surgical management outcomes of patients with mechanical strabismus and diplopia as a complication of FESS in a series of consecutive cases.

## 2. Material and Methods

The clinical records of all patients referred to our countrywide referral strabismus center over the 5-year period from 2018 to 2023 due to troublesome diplopia occurring after FESS were retrospectively reviewed. FESS procedures were performed at various clinics.

The collected data consisted of the patients’ sex, age, previous ocular history (especially the presence of childhood strabismus), as well as the type of FESS performed and the indications for surgery. Complete ophthalmic and orthoptic examinations were performed in each patient. The strabismus deviation was measured at distance and near using the prism alternate cover test in nine cardinal positions of gaze. Additionally, measurements on synoptophore were performed. Any abnormal head posture or lid anomalies were also noted. Computed tomography (CT) and/or magnetic resonance imaging (MRI) scans of the orbits were performed in each patient before surgical intervention. Any findings, such as orbital wall damage, extent of injury to the muscle or surrounding tissues, and enophthalmos, were evaluated both by an experienced radiologist and a strabismic surgeon. The type and timing of strabismus surgery were analysed in all patients. At postoperative orthoptic 6-month evaluations, the necessity of further interventions and the outcome were noted.

All procedures were performed in compliance with relevant laws and institutional guidelines. The Ethical Committee of Medical University of Lodz approved the study and data-gathering protocol (number RNN/47/24/KE consent of the day 13 February 2024). Informed consent was obtained from all of the patients.

## 3. Results

This case series comprised seven patients (five men, two women) with a mean age of 44.4 ± SD 4.29 years (range 40–50 years). The symptoms at presentation varied and depended on the time of referral, which ranged from one day (the day after FESS) to two months. All patients presented with troublesome diplopia and mild to severe abnormal head posture (Table 1).

Ipsilateral decreased visual acuity was reported by two patients (cases 2 and 4), one patient (case 1) had anisometropic amblyopia in the unaffected eye. One patient (case 2) had a transient decrease in visual acuity that correlated with subconjunctival oedema and hematoma with complete recovery within days.

Enophthalmos was observed in two patients (cases 3 and 7), in whom large fat compartment displacement or herniation of other soft tissue was revealed. Eyelid swelling and subconjunctival haemorrhage were reported only in the cases referred during the first two weeks after complicated FESS (cases 2, 4, 6, and 7). Increased intraocular pressure (IOP) was observed in one patient (case 2) and responded well to topical IOP-lowering eye drops (timolol and dorzolamide).

Detailed information concerning clinical picture of the studied patients is presented in Table 2. All patients had unilateral extraocular muscle injury, proven by CT or MRI scan.

**Figure 1:**
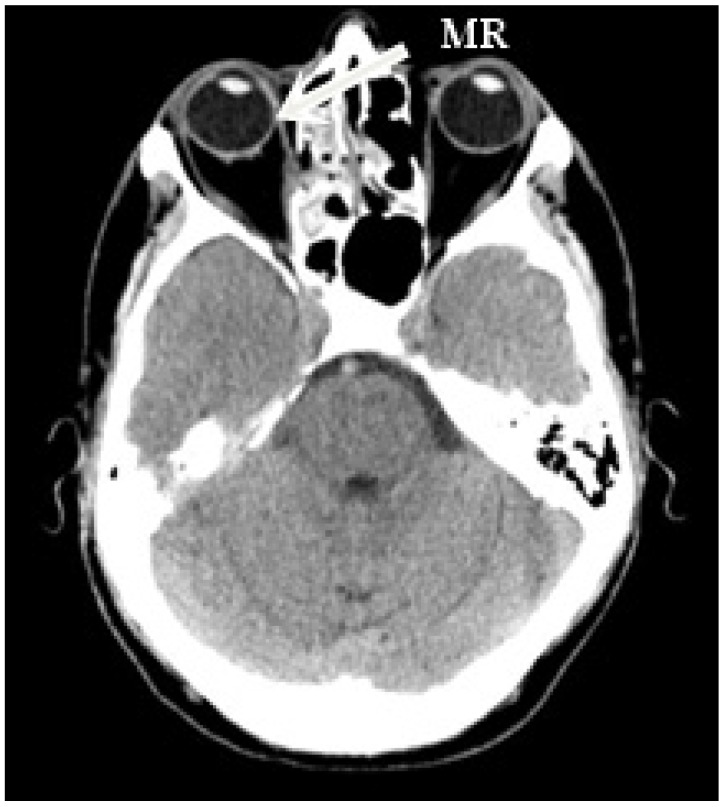
Patient 7. Axial computed tomography scan demonstrating defect in right medial orbital wall. The medial rectus was displaced medially and found adherent to the affected site, partially entrapped with defect in the lamina papyracea.

**Figure 2:**
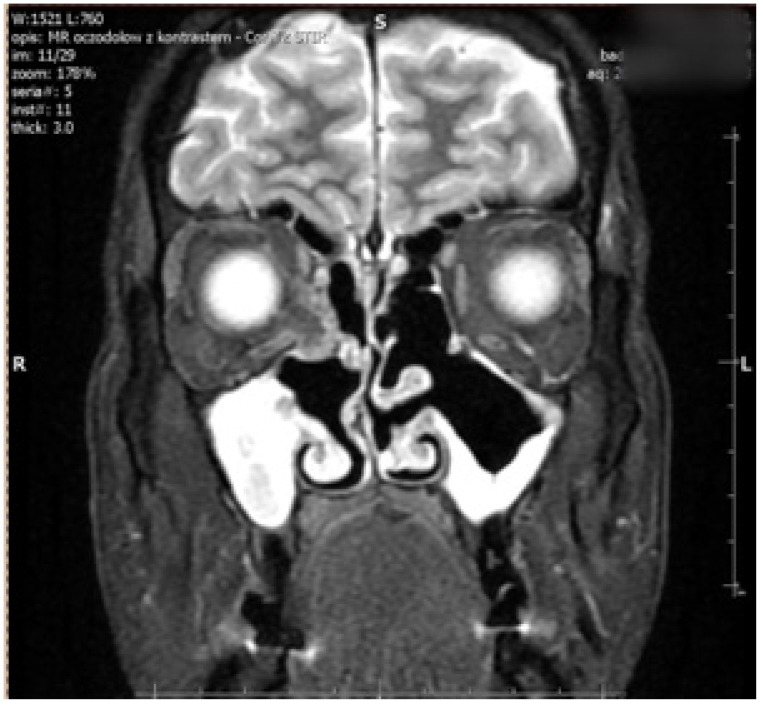
Postoperative coronal MRI scan showing bilateral ethmoidectomy, antrostomy, and uncinectomy in chronic maxillary sinusitis with significant mucosal thickening. MRI scan demonstrates the right medial rectus displacement.

The right side was more commonly affected (five right, two left) (Figure 1). The medial orbital wall was fractured in all but one case (case 5) where the damage involved the inferior wall. Prolapse of the orbital fat and soft tissue into the field of injury were described in all patients who experienced medial wall defect. Air-filled cavities were commonly seen around the injury and the characteristic lesions for conducted endoscopic surgery were visible on all scans (Figure 2).

The medial rectus muscle was most commonly involved and its injury was especially associated with ethmoidectomy. Destruction of the MR was observed in four cases (1–4) and partial destruction in one case (case 6). In one case, the MR was displaced medially and found adherent to the affected site, partially entrapped (case 7). In cases 5 and 7, contusion and oedema of the muscle were observed, while muscle continuity was preserved. One patient was found to have simultaneous SO impairment in addition to the medial rectus injury (case 3), another demonstrated isolated injury to the IR during antrostomy (case 5). In one patient, there was a risk of simultaneous injury to MR and IR, but that was not confirmed during imaging, nor intraoperatively (case 2).

Large-angle exotropia with severely limited or absent adduction was the most frequently observed pattern of motility disorders. Vertical deviation was also present in four patients (case 1, 2, 5, and 6). In three of these (case 1, 2, and 5) the hypertropic eye was ipsilaterally to the affected side and contralaterally in one case (case 6).

One patient underwent neurosurgical intervention with an unsuccessful attempt to reconnect the MR stumps via transnasal access (case 3); in this case, to obtain stabilisation of the eye globe, an intraoperative decision was made to leave the proximal stumps herniated into the fracture. In one case, orbital reconstructive surgery was performed with a titanium mesh implantation (case 7), resulting in improved ocular motility and a noticeable reduction of enophthalmos.

All except one patient required surgical correction of introgenically induced strabismus. The operations were performed by three surgeons. Each patient underwent a different strabismic, surgical approach, depending on the type of muscle injury, the time of referral, and outcomes of forced duction test (FDT). All procedures were performed only on the affected eye under general anesthesia. The details regarding the type of surgery performed in each patient are available in Table 2. In five cases, a second strabismic surgery was necessary. Surgical success was considered as no diplopia in the primary position. The follow-up ranged from six months to two years.

Direct reattachment of the MR ends was possible only in two patients, with a satisfactory result achieved only in one of them (case 2); the other patient (case 6) required secondary procedure involving recession of the LR. Both patients were referred immediately after the complicated FESS procedure, i.e. within one to three days. In other cases, if the adhesions had formed, the revision of the impaired muscle was conducted and encountered lesions were removed. A two-stage surgical strategy was planned for patients at increased risk of anterior segment ischemia. In patients with total damage of the extraocular muscle the transpositions procedures or permanent fixation to periosteum were recommended.

**Figure 3:**
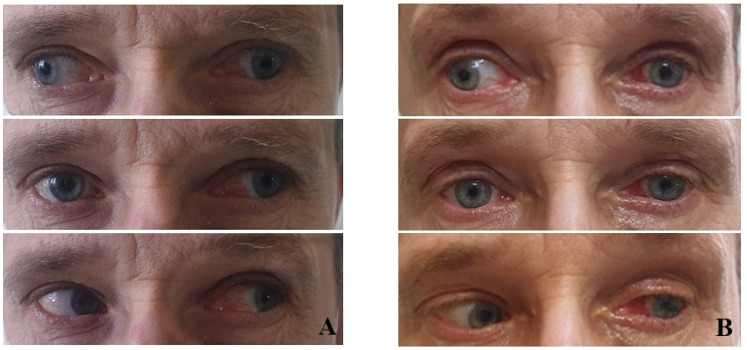
Patient 3. with simultaneous medial rectus injury with additional superior oblique impairment. (**A**) Preoperative photographs show left exotropia of 60 PD increasing on right gaze and decreasing on left gaze. (**B**) Postoperative photographs show satisfactory primary position alignment.

The FDT was completely free in three patients (cases 2, 6, and 7). In all other cases, who referred later than two weeks after the FESS procedure, prominent limitations were observed. Restrictions caused by scarring and fibrosis adjacent to injured muscle limited FDT movement, not only in the field of its action, but also in other directions. In three cases (1, 3, and 4), excessive adhesions (tissue conglomerates—bone, fat, and muscle) were encountered, which made EOM isolation challenging. In one of these cases, permanent periosteal fixation of the globe was performed (case 4). It involved the use of Tutopatch implant as a globe retractor (Figure 2) In case 3, massive adhesions around MR and SO had to be removed to mobilise the eye rotations; however, this inadvertently implied partial removal of the muscle themselves. To compensate for the deficient adduction and overelevation in adduction, which inevitably resulted from this procedure, LR recession of 13.0 mm and IO recession of 14.0 was performed. Due to the increased risk of anterior segment ischemia, a second surgical procedure was planned for six months later. During the procedure, the halves of the superior and inferior rectus muscles were transposed to the insertion of MR with a Foster augmentation suture; the effect was satisfactory—an exophoria (Figure 3).

Patient 1 had initially undergone a Nishida transposition procedure augmented with large (12 mm) recession of LR. The outcome was poor and a second intervention involving Y-splitting of the LR was performed.

In case 5, revision of IR and MR was performed, and the adhesions were found and freed. Although this procedure reduced horizontal misalignment significantly, it had a mild impact on the vertical deviation. This patient was advised to consider further surgical intervention, but was lost to follow up.

## 4. Discussion

A variety of injuries to EOM may occur after complicated FESS. The proximity of paranasal sinuses to the orbit and its contents increases the risk of their damage, especially when anatomical variations exist. This makes FESS challenging, especially to an inexperienced surgeon. Some varieties of the procedure are more risky than others: for example, surgery of the ethmoid sinuses is associated with a greater risk [7]. The lamina papyracea (LP) separates the ethmoid labyrinth from the contents of the orbit. Worden at el. found the mean distance between the LP and MR to be 3.6 mm at the level of the anterior aspect of the anterior ethmoid and 1.5 mm at the level of the basal lamella [8]. This close proximity implicates a greater risk of MR injury. The skills of the surgeon and the gravity of the pathology also seem to be obvious correlations, as well as the overall condition of the patient, presence of comorbidities, and history of previous surgeries [1].

Most commonly encountered complications can be regarded as minor, major, or serious [6], and classified into four categories based on the affected structure: orbit, optic nerve, lacrimal drainage system, or EOM [9]. Most ophthalmic complications of FESS are trivial, and their frequency varies among studies, ranging from 0.4% to almost 30% [5,6,10]. Major complications have been noted in 0.01 to 2.25% of cases [10]. In the present study, complications on the right side were more prevalent (71% right to 29% left), which can be partly explained by the position of most right-handed surgeons [6]. The visualisation and device manipulation are more difficult when right-handed surgeon is approaching the right side.

Among the various orbital complications, like enophthalmos, lipogranuloma formation, or orbital emphysema, the most serious is orbital hematoma. Severe retrobulbar haemorrhage can lead to compressive optic nerve neuropathy and is a sight-threatening condition. Direct optic nerve damage may occur during an attempt to remove Onodi cells, the posterior ethmoid air cells alongside the optic canal [7], or more frequently, following misdirection of the endoscopic instruments into the orbit [7].

One of the most debilitating complications is the injury to the EOM, which may induce significant ocular motility impairment, diplopia, and compensatory head posture. The damage to the muscle might be indirect or direct, by plain transection or excision of the part of the muscle belly. The latter is usually related to muscle ischemia [11] or dysinervation which results from the injury to the vessels and nerves supplying the EOM. Both mechanisms lead to a paretic form of strabismus.

Ocular motility impairment and diplopia are strongly suggestive of the damage to EOM. The most susceptible to direct injury is MR muscle, especially when mechanised systems are used for sinus debridement [7]. The microdebrider undoubtedly has revolutionised FESS surgery by providing a precise control and real-time visualisation of removing tissues and minimising intraoperative bleeding. However, it provides only minimal tactile feedback, especially during the removal of soft tissue, which can contribute to rapid and irreversible devastations [12]. Ethmoidectomy and uncinectomy are most commonly associated with medial orbital wall damage, while maxillary antrostomy and sinusotomy induce inferior orbital wall injury [13]. The LP is the thinnest of the seven bones of the orbit. When the papyraceous membrane is injured, there is a great risk of injury to the MR caused by sucking orbital fat [6]. Many types of injury to the muscles are possible depending on the extent, such as contusion, disruption, laceration, or the most serious: transection. However, the most commonly traumatised muscle in our series was the MR; in two cases, we found SO and IR damaged (cases 3, 5).

A very high correlation has been reported between MRI findings and clinical manifestations, such as abnormal eye movements or abnormal eye position. Gadolinum-enhanced MRI is recommended for the precise detection of injury to the EOM. CT is not a suitable test for detecting oedema in the acute or subacute stage; in some cases, the injury can be wrongly diagnosed [14]. As imaging provides information about the side, extent, and type of injury, the detailed imaging before strabismic reconstructive surgery is crucial.

CT is dedicated to the evaluation of the bony defects or bony entrapment of the EOM, while MRI is very useful for evaluating the entrapment of intraorbital fat or EOM into the sinuses [14]. However, it should be emphasised that orbital endoscopic penetration can occur without a radiologically detectable bony defect. Sometimes, small bony defects of the orbital walls are created during the FESS procedure without entering the orbit itself [13]. They are not visible in CT scans, but are enough for orbital fat or muscles to be aspirated into the sinus cavity [13]. Moreover, as orbital content passes back and forth through the defect, injuries to the EOM and its surroundings may still appear normal on CT scans [13].

Both CT and MRI play an essential role in surgical planning. The risk of inadvertent entry to the orbit can be minimised by good preoperative assessment [7]. In more complicated cases, Thacker recommends the use of multipositional MRI, which can detect muscle contractility [15]. The procedure is similar to that used in patients after blow-out fracture of the orbit and enables differentiation between restrictive and paretic form of motility disorders [16].

Regardless of the type of injury, systemic corticosteroids should be applied in the early postoperative period to reduce inflammation and scarring [17]. Clinically evident enopthalmos or muscle entrapment confirmed in MRI imaging requires orbital exploration and release of the entrapped tissues [7]. If there is no entrapment or direct trauma to EOM, the watch-and-wait approach for spontaneous improvement is recommended; such ocular motility impairment may be of paretic origin, which may resolve without intervention. It has been suggested that botulinum toxin should be injected into the antagonist muscle in the early postsurgical period to minimise its evolving contracture [17].

If imaging indicates direct muscle injury in the form of transection or partial excision, surgical reintervention should be considered. When the posterior portion of the transacted muscle is retraced posteriorly, Thacker advocates an orbital surgical approach [15]. The cut muscle can be retrieved by an anterior orbital approach; however, this should be only considered if the posterior portion of MR is longer than 20 mm and has evident function indicated by multipositional MRI [15]. The location of the transection is important because of the nerve supply: there is a branch of the oculomotor nerve sent to MR between the posterior and middle third of the muscle’s length [18]. When total muscle transection occurs, an evaluation of the distance between the proximal and distal stumps of the muscle helps to predict the possibility of direct reattachment.

In our series, primary reattachment of MR was performed in two patients, achieving good ocular alignment, although with decreased adduction (cases 2 and 6). Most importantly, direct reattachment was only possible in those patients who were referred early. The presented series of patients shows that early recognition is a greatly important factor in successful treatment. Some authors compare the repair of the myectomised muscle to retrieving a slipped or lost muscle [18], when the muscle remnants can be attached to the periorbita or other tissues; however, it is more difficult, considering the surrounding damage. Bone fragments and excessive bleeding from the sinus are also an obstacle.

In some cases, inadvertently myectomised muscle requires the interposition of the defect, since direct reattachment is not possible. This could be achieved by a hang-back suture proposed by Trotter [18] or with the use of muscle elongation materials, such as a silicone expander or Tutopatch [19,20]. The latter was successfully used in one our patients, but in a different manner: i.e., to achieve permanent fixation of the eye globe (case 4).

In more severe cases, when direct reattachment is not possible or when the muscle’s function is entirely lost, a transposition procedure should be considered [15]. In some cases, this procedure cannot be performed as a first-line therapy due to the massive adhesions adjacent to EOM. The muscle remnants and connective tissue proliferations have to be removed, and a large recession of the ipsilateral antagonist is the most that can be done. Transposition of the vertical rectus muscles should be postponed in order to avoid the risk of anterior segment ischemia (case 3). Full-tendon or half-tendon width transposition can be augmented by the posterior fixation sutures, described by Foster [21]. Vessel-sparing transposition of the vertical muscles is reserved for elderly patients with cardiovascular risk factors. Kong et al. describe two cases of successful treatment of large-angle exotropia with free FDT (−80 PD and −90 PD in primary position), in which a modified Nishida transposition and ipsilateral LR recession were performed [22]. This procedure is based on the redirection of force vectors to support weak muscle action; however, it will not work if a positive FDT is present. Therefore, the Y-splitting transposition of LR was used as a second-stage procedure in the present study; this allowed for diplopia to be eliminated in the primary position (case 1). In our present case series, three types of transposition procedures were performed in two cases as a primary or secondary procedure (cases 1 and 3).

Total loss of EOM function is a considerable problem, but the orbital scarring that results from a damage to the orbital fat pad and the Tenons capsule that surrounds the globe is a much greater issue. It complicates not only the surgery, but also the diagnostic process. Primarily, muscle damage limits the duction of the eye in the direction of its action, as in paretic strabismus. However, adhesion formation, especially between the muscle’s surroundings and the orbital periosteum limits duction in the opposite direction. For example, if the MR muscle is damaged and adhesions are formed, the eye is not able to adduct because the muscle is transected, nor abduct because the scar restricts its movement. The severity of duction limitation is related directly to the amount of orbital fat that has slipped into the Tenon’s space between the muscles and the globe surface. In rare cases, excessive scarring can lead to a ‘frozen orbit syndrome’–globe external restrictive ophthalmoplegia [13].

As such, FDT plays an essential role in the diagnostic process in cases of post-FESS orbital complications. It must be performed at the beginning of strabismus surgery, and the surgical plan should be altered according to the findings. Scarring and adhesions sometimes expand heavily, surpassing the site of the lesion itself [13]. The surgeon should be aware of the possibility of orbital fibrosis or diffused intraorbital fat before the procedure begins, as this may affect the surgical plan. When a patient is cooperative, the test can be performed in an outpatient setting, along with the active force generation test, which gives an assumption of the paretic muscle remnant force. The latter gives an indication of how much tonus is left in the injured muscle and allows the surgeon to tailor the transposition surgery accordingly to avoid overcorrection. In the studied group, FDT yielded valuable information, especially in those patients where massive adhesions were present.

Sometimes, the presence of extensive fibrosis or fat adherence syndrome requires removal of the adhesions and freeing the globe as the first step to prepare the field for further surgeries. This happened in case 3, where extensive fibrosis of MR and SO occurred. In such cases, as the result of the first stage of the surgery can be uncertain, transposition surgery should be postponed. Also, the risk of anterior segment ischaemia requires surgical plans to be modified or postponed for at least six months. As such, patients with strabismus after FESS should be always informed that surgical correction might involve several stages.

This study has several limitations. Firstly, it is a retrospective study, which relies on existing clinical records, rather than a prospective, standardised data collection. Secondly, the sample size was relatively small as the complication is extremely rare. Lastly, the study only included patients with more severe or complex post-FESS diplopia who were referred to our centre, recognised as the top referral strabismus centre in Poland. This may have led to a biased sample population, as it focused only on individuals with significant difficulties and excluded those with milder symptoms or who sought care elsewhere.

## 5. Conclusions

The FESS technique has significantly improved during the last decades. EOM injuries are rare, but may leave the patient severely disabled due to diplopia and motility limitation associated with a compensatory head posture. A meticulous preoperative assessment including accurate evaluation of imaging along with FDT should be performed in all cases, as the findings can allow for a better determination of the surgical approach and prepare the surgeon for potential intraoperative changes in the surgical plan.

Early intervention was related to the better outcomes. Fat adherence syndrome or adhesion creation and scarring generate restricted ductions in more than one direction, making surgery and proper ocular alignment more challenging.

The surgical technique should be tailored to each individual case, and the final plan depends on the severity, type of injury, number of muscles involved, and FDT. Primary reattachment should be considered when only one muscle is damaged, and its stumps are long enough and functional. In more severe injuries with significant muscle destruction, transposition techniques should be considered. In patients with excessive scarring, especially posterior to the equator, treatment options are limited and usually permanent fixation to the periosteum is feasible. Patients should be always informed that more than one strabismic operation will probably be necessary in order to reduce troublesome diplopia.

## Figures and Tables

**Table 1:** Type of endoscopic surgery, side and nature of the injury-extraocular muscle involvement, and preoperative findings (eye motility, head turn, other ocular findings).

Patient No.	Type of FESS Surgery	Side of Injury	Type of Injury	Preoparative Eye Motility	Head Turn	Other Ocular Findings
1	Bilateral ethmoidectomy	Right	Medial orbital wall fracture Transection of MR	No adduction (−5)	30°	Anisometropic amblyopia
2	Bilateral antrostomy	Right	Medial orbital wall fracture Transection of MRFat prolapse	Decreased adduction (−2)	10°	↓ VA 16/20 ↑ IOP Eyelid hematoma, eyelid swelling, subconjunctival hemorrhage
3	Bilatetral frontethmoidectomy	Left	Medial orbital wall fracture MR, SO disruption, MR-entrapment Fat prolapse	No adduction (−5), slighlty impaired depression (especially depression in adduction	35°	Enopthalmos
4	Unilateral ethmoidectomy	Left	Medial orbital wall fracture MR disruption + entrapment Fat prolapse	Limited adduction (−3)	25°	↓ VA 14/20 Eyelid hematoma, eyelid swelling, subconjunctival hemorrhage
5	Bilateral antrostomy	Right	lnferior orbital wall fracture IR contusion	Limited downgaze (−2)	No	-
6	Bilateral ethmoidectomy	Right	Medial orbital wall fracture MR incomplete destruction + fat prolapse	Limited adduction (−3)	20°	Hematoma
7	Bilateral ethmoidectomy	Right	Medial wall fracture-ethmoid bone defect, MR contusion + MR entrapment + fat prolapse	Limited abduction (−3)	No	Enopthalmos

**Table 2:** Timing from FESS to surgery, preoperative alignment, summary of surgical procedures (with FDT findings), and the outcomes in patients after complicated FESS.

Patient No.	Time From FESS To Surgical Procedure	Preoperative Deviation in the Primary Position	FDT	Type of 1st Strabismic Surgery	Postoperative Deviation	Type of 2nd Strabismic Surgery	Posoperative Deviation II
1	2 months	−60 PD R/L 5 PD	Limited adduction	OD: recesssion LR 12.0 mm + Nishida transposition of vertical muscles to MR	−40 PD	OD: Y splitting transposition LR	−4 PD P/L 2 PD
2	1 day	−16 PD R/L 6 PD	Free	OD: direct reattachment	0 PD R = L	NO	NO
3	1 month	−60 PD	Limited adduction, elevation in adduction, abduction	OS: massive adhesions around MR and SO removal-> SO tenectomy LR Recession 13.0 mm IO recession 14.0	−40 PD L/R 5 PD	OS: Hummelsheim vertial muscles transposition to MR + Foster sutures	−4 PD
4	2 weeks	−30 PD	Limited adduction	OS: attempt of reattachment of distal and proximal parts of MR—unsuccessful-> MR proximal part (1 cm) was sawn to the medial orbital periosteum	−30 PD	OS: Tutopatch insertion between proximal and distal part of MR	−14 PD
5	2 months	−8 PD R/L 10 PD	Limited depression + elevation	OD: IR, MR revision	0 PD R/L8	NO	-
6	3 days	−30 PD L/R 5 PD	Free	OD: direct reattachment	−15 PD	OD:LR recession 8 mm	−4 PD
7	4 days	0 PD R = L	Free	None	-	-	-

## Data Availability

Data are contained within the article.

## Abbreviations

CT	Computed tomography
FDT	Forced duction test
FESS	Functional endoscopic sinus surgery
EOM	Extraocular muscles
IO	Inferior oblique
IR	Inferior rectus
LP	Lamina papyracea
LR	Lateral rectus
MRI	Magnetic resonance imaging
MR	Medial rectus
SO	Superior oblique
SR	Superior rectus
